# Resveratrol Regulates BDNF, trkB, PSA-NCAM, and Arc Expression in the Rat Cerebral Cortex after Bilateral Common Carotid Artery Occlusion and Reperfusion

**DOI:** 10.3390/nu11051000

**Published:** 2019-05-01

**Authors:** Maria Pina Serra, Marianna Boi, Laura Poddighe, Tiziana Melis, Ylenia Lai, Gianfranca Carta, Marina Quartu

**Affiliations:** Department of Biomedical Sciences, University of Cagliari, Cittadella Universitaria, 09042 Monserrato, Italy; mpserra@unica.it (M.P.S.); marianna.boi@unica.it (M.B.); laura.poddighe@gmail.com (L.P.); tizianasilem@yahoo.it (T.M.); ylenia.lai@gmail.com (Y.L.); giancarta@unica.it (G.C.)

**Keywords:** resveratrol, dietary supplements, BDNF/trkB, PSA-NCAM, immediate early genes, Arc protein, acute bilateral common carotid artery occlusion, cerebral cortex, Western blot, immunohistochemistry

## Abstract

The polyphenol resveratrol (RVT) may drive protective mechanisms of cerebral homeostasis during the hypoperfusion/reperfusion triggered by the transient bilateral common carotid artery occlusion followed by reperfusion (BCCAO/R). This immunochemical study investigates if a single dose of RVT modulates the plasticity-related markers brain-derived neurotrophic factor (BDNF), the tyrosine kinase trkB receptor, Polysialylated-Neural Cell Adhesion Molecule (PSA-NCAM), and Activity-regulated cytoskeleton-associated (Arc) protein in the brain cortex after BCCAO/R. Frontal and temporal-occipital cortical regions were examined in male Wistar rats randomly subdivided in two groups, sham-operated and submitted to BCCAO/R. Six hours prior to surgery, half the rats were gavage fed a dose of RVT (180 mg·kg^−1^ in 300 µL of sunflower oil as the vehicle), while the second half was given the vehicle alone. In the frontal cortex of BCCAO/R vehicle-treated rats, BDNF and PSA-NCAM decreased, while trkB increased. RVT pre-treatment elicited an increment of all examined markers in both sham- and BCCAO/R rats. No variations occurred in the temporal-occipital cortex. The results highlight a role for RVT in modulating neuronal plasticity through the BDNF-trkB system and upregulation of PSA-NCAM and Arc, which may provide both trophic and structural local support in the dynamic changes occurring during the BCCAO/R, and further suggest that dietary supplements such as RVT are effective in preserving the tissue potential to engage plasticity-related events and control the functional response to the hypoperfusion/reperfusion challenge.

## 1. Introduction

Increasingly emerging evidence indicates that natural products with anti-oxidative and anti-inflammatory properties may prevent and counteract the hypoperfusion/reperfusion tissue challenge induced by acute transient bilateral common carotid artery occlusion followed by reperfusion (BCCAO/R) [1,2,3,4]. As a model of global hypoperfusion/reperfusion, the acute transient BCCAO/R can be used to investigate *in vivo* the biological effects of acute cerebral oxidative stress and the early formation of a deleterious pro-inflammatory milieu. BCCAO/R-induced derangement of brain tissue physiological homeostasis can be directly correlated to molecular changes that can be found in brain tissue and plasma as early as 30 min [5] to 2.5 h after surgery [1,2,6,7,8].

A number of independent studies have contributed to develop and consolidate the notion that dietary control and nutritional intake are potent environmental regulators of brain neuroplasticity in health and well-being [9]. Resveratrol (RVT) (3,4′,5-trihidroxystilbene) is a natural polyphenol found in different vegetal species, grapes and red wine [10,11], produced naturally by some plants in response to injury or pathogens [12]. RVT has manifold beneficial properties for cerebral tissue homeostasis, related mainly to its ability to scavenge the free radicals by inducing anti-oxidant pathways and exerting anti-inflammatory effects [10,13,14,15]. Preclinical studies have shown the RVT therapeutic properties against ageing [10], chronic inflammation [16,17], cardiovascular syndromes and neurological disorders [10,11,18,19,20], and in models of transient and chronic ischemia [21,22,23]. Many studies have added to the knowledge of mechanisms underlying the responsiveness of intracellular signaling transducers to RVT [24]. Thus, during stroke [25] and in the BCCAO/R model [4], RVT-induced neuroprotection appears to be directly correlated to the peroxisome proliferator-activated receptor-α (PPAR-α), a lipid-sensing transcription factor through which RVT may control the expression of pro-inflammatory molecules in the nervous system [26,27,28,29,30]. Parallel studies on animal models *in vivo* have further unveiled effects of RVT in neuroprotection [13,31,32], including its ability to revert synaptic plasticity deficits following acute [4,33] and chronic cerebral hypoperfusion [34]. Accordingly, in the BCCAO/R model, RVT pre-treatment restores the tissue concentration of docosahexaenoic acid (DHA), a structural component of neural cell membranes, and increases expression of synapse-associated proteins [4]. In this context, it is not surprising that RVT has also been shown to modulate the expression of brain-derived neurotrophic factor (BDNF) [35,36,37,38], a member of the neurotrophin family that signals through the tyrosine kinase receptor B (trkB). The BDNF-trkB system plays an instructive role for synaptic plasticity [39], involving activation of cytoskeleton dynamics in dendritic spines. Accordingly, BDNF is implicated in local translation of the Activity-regulated cytoskeleton-associated (Arc) protein [a product of the immediate early gene (IEG) *Arc*] [40,41]. Additionally, modulation of neuronal signaling is supported by growth-associated proteins, such as the polysialylated form of the neural cell adhesion molecule (PSA- NCAM) [42], which facilitates BDNF-trkB interaction [43]. This study, carried out by means of Western blot analysis and immunohistochemistry, had a twofold aim. Firstly, to further characterize the neuronal molecular substrates possibly involved in cerebral hypoperfusion/reperfusion-induced tissue damage and especially in synaptic adaptive processes [4], we investigated on possible BCCAO/R-induced changes in the plasticity-related markers BDNF, trkB, PSA-NCAM, and Arc protein. Secondly, we investigated the ability of a single acute dose of RVT to prevent the BCCAO/R-induced changes in neuroplasticity markers. For this research we aimed to examine the forebrain areas that, being directly and selectively reached by terminal branches of the internal carotid artery, appear to be particularly affected by the BCCAO/R [1,3,4,6], and the temporal-occipital cortical regions that, being supplied by ramifications of the basilar artery, can be used as a control. Results are discussed in light of the possible significance of BDNF-trkB system, PSA-NCAM and Arc as early markers of a transient cerebral global hypoperfusion, the role of RVT as regulator of neuroplasticity, and the prospect to employ RVT as a dietary supplement to control the physiological adaptive response to the cerebral hypoperfusion/reperfusion.

## 2. Materials and Methods

### 2.1. Animals and Keeping

Adult male Wistar rats (Harlan, Udine, Italy), weighing 210 ± 20 g (mean ± SD), for 1 week before the experiment begun were housed under controlled temperature (21 ± 2 °C), a 12 h light/dark cycle, and relative humidity (60 ± 5%), avoiding any distress of animals. Rat handling and care met with national (Legislative Decree n. 26, 04/04/2014) and international (Directive 2010/63/EU) laws and policies. The experimental protocols conformed to the guidelines of the Animal Ethics Committee of the University of Cagliari (approval code No. 06/2013, 05/31/2013). Standard laboratory food (A04, Safe, Augy, France) and water were freely available ad libitum.

Animals were not fed for 12 h before surgery. Rats (*n* = 46) were randomly assigned to two groups that, 6 h before the surgery, were gavage fed a pre-treatment: one group (vehicle-treated) received the vehicle, i.e., 0.3 mL of sunflower oil, while the other (RVT-treated) was given 40 mg of RVT (Tokyo Chemical Industry Co., Portland, OR, USA) (equivalent to 180 mg·kg^−1^), dissolved in 0.3 mL of sunflower oil as the vehicle. The choice to use a vehicle was made to guarantee that an amount of RVT as accurate as possible could be administered via gavage and to facilitate the absorption of RVT. Each group was subdivided into sham-operated or submitted to BCCAO/R.

### 2.2. Surgery

The surgical procedure was adapted from the method of Iwasaki et al. [44] and, for all animals, performed between 13:00 and 16:30 p.m. An intraperitoneal injection of Equithesin (16.2% w/w pentobarbital, 4.2% w/v chloral hydrate, 39.6% w/w propylene glycol, 2.12% w/v MgSO4, and 10% w/w ethanol in sterile distilled H_2_O) (5 mL/100 g bodyweight) was used as anesthesia. A midline incision of the neck was followed by blunt muscle dissection to expose the common carotid arteries (CCA), taking care to leave the vagus nerve unhurt. To reduce the cerebral blood flow, the CCA were clamped for 30 min with 2 atraumatic microvascular clips. The reperfusion period was attained by removing the clips and restoring blood flow for 60 min. The sham-operated rats underwent surgery without CCA occlusion and for this reason they represented the control animals, used to determine the effects of anesthesia and surgical manipulation on the results.

### 2.3. Sampling

At the end of the BCCAO/R procedure, brain samples were collected either as fresh tissue for Western blot or after fixation by transcardial perfusion with ice cold fixative solution (0.1 M phosphate buffer (PB), pH 7.3 and 4% paraformaldehyde) for immunohistochemistry. For Western blot analysis, the cerebral cortex was rapidly dissected from the rest of the brain and transversely cut, respectively, at the approximate bregma level of −1.0 mm [45] for the frontal cortex, and of −4.5 mm for the temporal-occipital cortex, used as a control cortical area not supplied by the internal carotid artery branches; specimens were then frozen at −80 °C until analysis. For immunohistochemical assays, brains were post-fixed by immersion in freshly prepared fixative, pH 7.3, for 4–6 h at 4 °C, and then rinsed in 0.1 M PB, pH 7.3, containing 20% sucrose. For both Western blot and immunohistochemical assays, the investigator was blind with respect to the experimental condition of rats.

### 2.4. Western Blot

**Figure 1:**
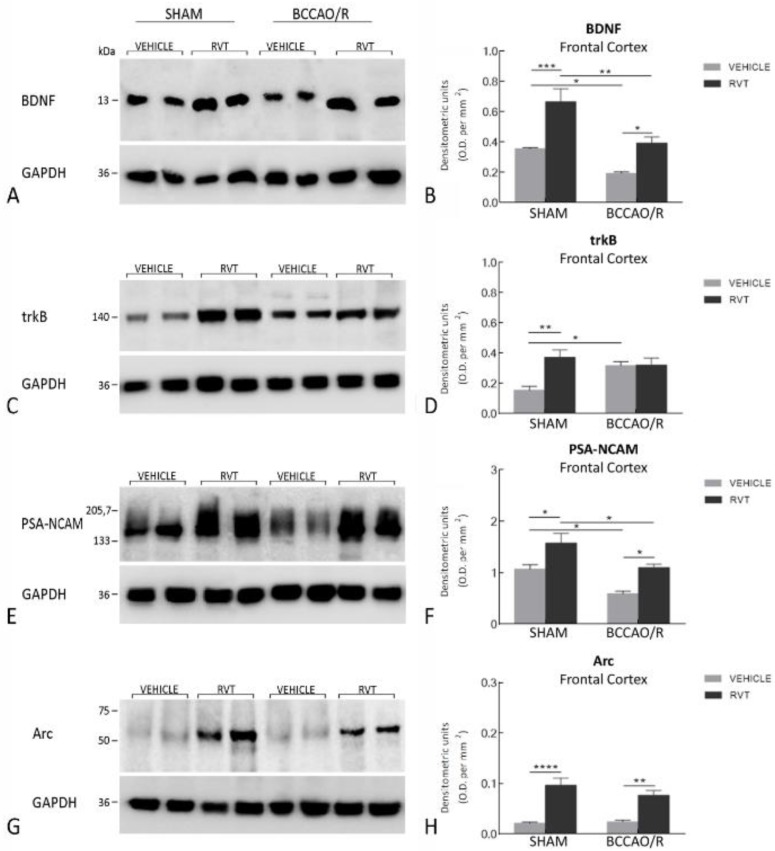
Western blot analysis of the frontal cortex homogenates in vehicle-treated and resveratrol (RVT)-treated rats either in sham-operated or after bilateral common carotid artery occlusion followed by reperfusion (BCCAO/R). (**A**,**B**) Brain-derived neurotrophic factor (BDNF), (**C**,**D**) tyrosine kinase receptor B (trkB), (**E**,**F**) polysialylated neural cell-adhesion molecule (PSA-NCAM), and (**G**,**H**) activity-regulated cytoskeleton-associated (Arc) protein. (**B**,**D**,**F**,**H**) Densitometric analysis of the band gray levels expressed as a percentage of the optical density (O.D.) ratio of BDNF-, trkB-, PSA-NCAM-, and Arc-positive bands to those immunostained for GAPDH. Data are reported as the mean values of 12 vehicle-treated and 10 RVT-treated rats. Error bars depict the standard error of the mean (S.E.M.). Asterisks denote significant differences. Two-way ANOVA with the Tukey’s test for post hoc analyses was applied to evaluate statistical differences between groups. * *p* < 0.05; ** *p* < 0.01; *** *p* < 0.001; **** *p* < 0.0001 (see Table 1 for *F*- and *p*-values relevant to effects of BCCAO/R and RVT pre-treatment and to the interaction between them).

Tissue homogenates (*n* = 12 vehicle-treated and 10 RVT-treated rats were prepared in a 2% solution of sodium dodecyl sulfate (SDS) containing a cocktail of protease inhibitors (cOmplete, Mini Protease Inhibitor Cocktail Tablets, Roche, Basel, Switzerland). Protein concentrations were determined in keeping with the Lowry’s protein assay method [46], using bovine serum albumin as the standard. Proteins for each tissue homogenate (40 μg), diluted 3:1 in 4× loading buffer (NuPAGE LDS Sample Buffer 4×, Novex by Life Technologies, Carlsbad, CA, USA), were heated to 95 °C for 7 min and separated by SDS-polyacrylamide gel electrophoresis (SDS-PAGE) using precast gradient gel (NuPAGE 4–12% Bis-Tris Gel Midi, Novex by Life Technologies) in the XCell4 Sure Lock^TM^ Midi-Cell chamber (Life Technologies). Internal mw standards (Precision Plus Protein^TM^ WesternC^TM^ Standards, Bio-Rad, Hercules, CA, USA) were run in parallel. Two gels at a time were run for immunoblotting and Coomassie blue staining, respectively. Proteins for immunoblotting were transferred by electrophoresis on a synthetic membrane of polyvinylidene fluoride (Amersham Hybond^TM^-P, GE Healthcare, Little Chalfont, U.K.) using the Criterion^TM^ Blotter (Bio-Rad). Blots were blocked by immersion in 20 mM Tris base and 137 mM sodium chloride (TBS), containing 0.1% Tween 20 (TBS/T) and 5% milk powder, for 60 min, at room temperature (RT). The primary antibodies were polyclonal antibodies raised in rabbit against BDNF (Cat# N-20 sc-546, SCBT, Santa Cruz, CA, USA) and trkB (Cat# 794 sc-12, SCBT), both diluted 1:1000, and monoclonal antibodies raised in mouse against PSA-NCAM (Cat# MAB5324, EMD Millipore, Darmstadt, Germany), diluted 1:1000, and Arc (Cat# sc-17839, SCBT, Santa Cruz, CA, USA), diluted 1:300 in TBS containing 5% dried milk and 0.02% sodium azide. Incubations were performed at 4°C and lasted 48 h for BNDF, trkB, PSA-NCAM and 24 h for Arc. After rinsing in TBS/T, blots were incubated at RT, for 60 min, with peroxidase-conjugated goat anti-rabbit (Cat#9169, Sigma Aldrich, St. Louis, MO, USA), diluted 1:10,000, and anti-mouse serum (AP124P, Millipore, Darmstadt, Germany), diluted 1:5000 in TBS/T. Controls for equal-loading of the wells were obtained by immunostaining the membranes, as above, for glyceraldehyde-3-phosphate dehydrogenase (GAPDH), using a mouse monoclonal anti-GAPDH antibody (MAB374, EMD Millipore, Darmstadt, Germany) diluted 1:1000, as the primary antiserum, and a peroxidase-conjugated goat anti-mouse serum (AP124P, EMD Millipore, Darmstadt, Germany), diluted 1:5000, as secondary antiserum. To control for non-specific staining, blots were stripped and incubated with the relevant secondary antiserum. To test out antibody specificity and cross-reactivity, the anti-BDNF antibody was preabsorbed with 200 ng of rhBDNF (Cat# B-257, Alomone Labs, Jerusalem, Israel), while the anti-PSA-NCAM antibody was challenged with 500 ng of the alfa-2-8-linked sialic polymer colominic acid (Cat# sc-239576, SCBT, USA). Membranes were then rinsed in TBS/T, and protein bands revealed with the Western Lightning Plus ECL (Cat# 103001EA, PerkinElmer, Waltham, MA, USA), according to the manufacturer’s protocol, and visualized using the ImageQuant^TM^ LAS-4000 (GE Healthcare, Little Chalfont, UK). Approximate molecular weight (mw) and relative optical density (O.D.) of the immunostained protein bands were evaluated by a blinded examiner. Quantification of O.D. was performed by means of the Image Studio Lite Software (Li-Cor, http://www.licor.com/bio/products/software/image_studio_lite/). The ratio between the intensity of the bands labelled for BDNF, trkB, PSA-NCAM, Arc and those positive for GAPDH ones was used to compare the relative levels of protein expression in the four experimental conditions, and is shown as histograms in Figure 1.

### 2.5. Immunohistochemistry

Coronal serial sections (14 μm thick) of frontal and temporal-occipital cortex were cut with a cryostat, collected on chrome alum-gelatin-coated slides and processed by the avidin–biotin–peroxidase complex (ABC) and the indirect immunofluorescence (IIF) techniques. Brains of vehicle (*n* = 12) - and RVT-treated (*n* = 12) rats were processed in pairs on the same slide. For the ABC, slides were firstly treated with 0.1% phenylhydrazine in phosphate buffered saline (PBS) containing 0.2% Triton X-100 (PBS/T) to inhibit the endogenous peroxidase activity, then with 20% of normal goat serum (Cat# S-1000, Vector Labs Inc., Burlingame, CA, USA) for 1 h at RT. Rabbit polyclonal antibodies against BDNF (SCBT, Santa Cruz, CA, USA), diluted 1:400, and trkB (SCBT, Santa Cruz, CA, USA), diluted 1:500, were used as primary antibody. For the IIF, polyclonal antibodies raised in rabbit against BDNF (SCBT, Santa Cruz, CA, USA), diluted 1:100, in rat against Glial Fibrillary Acidic Protein (GFAP) (Cat# CBL411, Cymbus Biotechnology, Southampton, Hampshire, UK), diluted 1:600, and in goat against Iba1 (Cat# NC 100-1028, Novus Biologicals, Littleton, CO, USA), diluted 1:1000, were used as primary antiserum and incubations were run overnight at 4 °C. In the ABC method, biotin-conjugated goat anti-rabbit serum (Cat# BA-1000, Vector Labs Inc., Burlingame, CA, USA), diluted 1:400, was used as secondary antiserum. The reaction product was revealed with the ABC (Cat#G011-61, BioSpa Div. Milan, Italy), diluted 1:250, followed by development with the cromogen solution of 0.05% 3,3′-diaminobenzidine (Sigma Aldrich, St. Louis, MO, USA), 0.04% nickel ammonium sulfate and 0.01% hydrogen peroxide in 0.1 M PB, pH 7.3. In the IIF protocol, Alexa Fluor 488- or Alexa Fluor 594-conjugated donkey antisera against goat, rat and rabbit proteins (Invitrogen, Eugene, OR, USA), diluted 1:500, were used as secondary antiserum. All antisera and the ABC were diluted in PBS/T. Incubation with primary antibodies was carried out overnight at 4 °C. Incubations with secondary antiserum and ABC lasted 60 min and 40 min, respectively, and were performed at RT. Negative control preparations were obtained by incubating tissue sections in parallel with either PBS/T, alone, or (i) with the relevant primary antiserum pre-absorbed with an excess of the corresponding peptide antigen (Cat# sc-546P and sc-12 P, for the BDNF and the trkB, respectively, SCBT, Santa Cruz, CA, USA); (ii) or by omission of the primary antibody; or (iii) by substituting the primary antibody with normal serum. Slides were observed with an Olympus BX61 microscope and digital images were acquired with a Leica DFC450 C camera.

### 2.6. Statistical Analysis

Data from the four experimental conditions, i.e., vehicle-treated and RVT-treated sham animals, and vehicle-treated and RVT-treated BCCAO/R rats, are depicted in Figure 1 as the mean ± standard error of mean (S.E.M.). Two-way analysis of variance (ANOVA) (main factors: (a) RVT-treatment (i.e., vehicle- vs. RVT-treatment) and (b) BCCAO/R (i.e., sham-operation vs. BCCAO/R) was performed using Prism 6.01 for Windows (GraphPad Software, La Jolla CA, USA, www.graphpad.com). Wherever appropriate (i.e., p for the main factors and their interaction < 0.05), multiple pair-wise contrasts were made, and the multiplicity adjusted p-value for each comparison was calculated using Tukey’s post hoc test.

## 3. Results

### 3.1. Western Blot Assays

The effects of the BCCAO/R without and with preventive administration of RVT on the relative levels of BDNF, trkB, PSA-NCAM, and Arc proteins are summarized in Table 1 and shown in Figure 1. Statistical analysis of O.D. values of the immunostained protein bands was carried out by two-way ANOVA (main factors BCCAO/R and RVT) (Table 1) showed that BCCAO/R-induced molecular changes as well as the effect of the RVT pre-treatment were obvious in the frontal cortex, while no statistically-significant differences were observed in the temporal-occipital cortex.

#### 3.1.1. The BDNF Protein Levels

The antibody against BDNF labeled a protein band with a relative mw of about 13 kDa (Figure 1A), consistent with the reported mw of the protein monomeric form [47,48]. Assessment of the BDNF densitometric values by a two-way ANOVA (Table 1) revealed effects of BCCAO/R (*p* < 0.0001) and RVT (*p* < 0.0001). Pair-wise contrasts further showed that in the vehicle-treated animals a statistically significant decrease of BDNF relative protein levels, amounting to −46%, occurred in BCCAO/R vs. sham-operated rats (*p* = 0.0489). After the RVT pre-treatment, relative levels of the BDNF protein were notably increased in both sham-operated (+ 87%; *p* = 0.0003) and BCCAO/R rats (+ 103%; *p* = 0.0180) as compared to the vehicle-treated rats; nonetheless, a statistically significant BCCAO/R-induced decrease of BDNF relative protein levels was observed as compared to sham-operated rats (−41%; *p* = 0.002).

#### 3.1.2. The trkB Protein Levels

The anti-trkB antibody, raised against the biologically active receptor protein in its full-length isoform, recognized a protein band with a relative mw of about 140 kDa (Figure 1C), consistent with the reported mw of the receptor protein [48,49]. A two-way ANOVA performed to assess the densitometric values of the trkB protein bands (Table 1) showed an effect of RVT pre-treatment (*p* = 0.0065) and an interaction of BCCAO/R and RVT (*p* = 0.0087). Post-hoc contrasts showed that trkB relative protein levels increased by 105% (*p* = 0.0178) in vehicle-treated rats. The RVT pre-treatment induced an increment of about 140% (*p* = 0.0024) in the trkB relative protein levels of the sham-operated vs. the vehicle-treated animals. No trkB relative level changes occurred in the RVT-treated BCCAO/R vs. the vehicle-treated BCCAO/R rats.

#### 3.1.3. The PSA-NCAM Protein Levels

The antibody against PSA-NCAM recognized a single broad band (Figure 1E), in agreement with the expected mw of the protein [50,51,52,53]. A two-way ANOVA revealed a significant effect of BCCAO/R (*p* = 0.0003) and of RVT (*p* = 0.0002). Pair-wise comparisons showed that a statistically significant decrease of PSA-NCAM relative levels occurred in BCCAO/R vs. sham-operated rats in both the vehicle-treated (−45%; *p* = 0.02) and RVT-treated rats (−44%; *p* = 0.0344). In RVT-treated animals, the PSA-NCAM relative protein levels were noticeably higher than the vehicle-treated animals in both the sham-operated (+ 46%; *p* = 0.0176) and BCCAO/R rats (+ 28%; *p* = 0.0185).

#### 3.1.4. The Arc Protein Levels

The anti-Arc antibody recognized a single band (Figure 1G) with a relative mw of about 55 kDa (Figure 1D), in keeping with the expected mw [54]. A two-way ANOVA showed a highly significant effect of RVT (*p* < 0.0001). In the RVT-treated group, post-hoc contrasts showed an increment of Arc as high as 350% (*p* < 0.0001) in sham-operated, and 217% (*p* = 0.001) in BCCAO/R rats as compared to the vehicle-treated ones.

### 3.2. Immunohistochemistry

**Figure 2:**
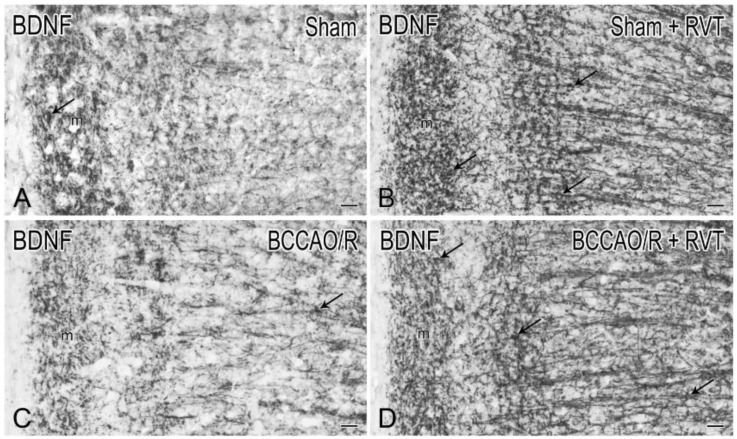
Brain-derived neurotrophic factor (BDNF)-like immunoreactivity in coronal sections of frontal cortex of sham-operated and bilateral common carotid artery occlusion followed by reperfusion (BCCAO/R) rats, pre-treated with either the vehicle alone (**A**,**C**) or with resveratrol (RVT) (**B**,**D**). Positive neuronal processes, punctate elements, and neuronal perikarya are distributed across the whole thickness of the cortex. Arrows point to immunostained neuronal perikarya. Panels are representative of observations carried out in 6 rats for each group. m, molecular layer. Scale bars: 25 μm.

**Figure 3:**
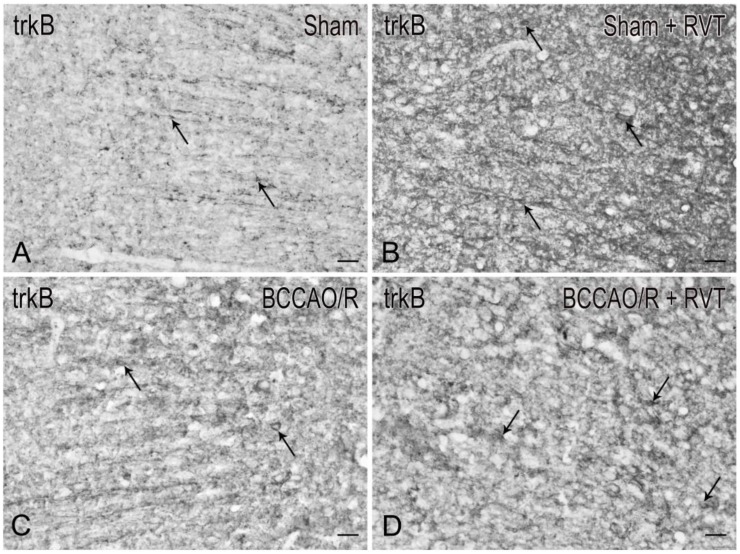
Tyrosine kinase receptor B (trkB)-like immunoreactivity in coronal sections of frontal cortex of sham-operated and bilateral common carotid artery occlusion followed by reperfusion (BCCAO/R) rats pre-treated with either the vehicle alone (**A**,**C**) or with resveratrol (RVT) (**B**,**D**). Positive neuronal processes, punctate elements, and neuronal perikarya are distributed across the whole thickness of the cortex. Arrows point to immunostained neuronal perikarya. Panels are representative of observations carried out in 6 rats for each group. Scale bars:  25 μm.

**Figure 4:**
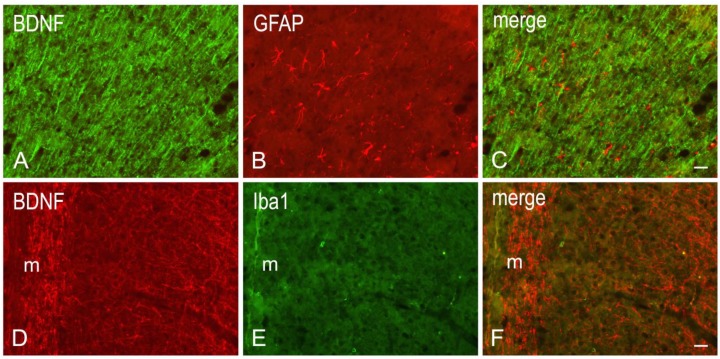
Double immunofluorescence for BDNF (**A**,**C**,**D**,**F**) and either glial fibrillary acidic protein (GFAP) (**B**,**C**) or Iba1 (**E**,**F**) in representative coronal sections of frontal cortex of BCCAO/R + RVT rats. m, molecular layer. Scale bars A, B = C: 25 μm; D, E = F: 25 μm.

In order to correlate the molecular changes observed by western blot analysis with the tissue morphology, the BDNF- (Figure 2) and the trkB-like immunoreactivity (LI) (Figure 3) were also studied in the cerebral cortex. Both BDNF- and trkB-LI were localized to neuronal structures distributed throughout the rostrocaudal extension of the frontal cortex and the temporal-occipital cortex. In the frontal cortex, BDNF- and trkB-immunoreactivity was localized to neuronal proximal processes and nerve fibers distributed throughout the cortical layers, having the aspect of loose networks of thin filaments and punctate elements in the superficial layers, and straight neuronal processes with a prevalent radial orientation in the deep layers. BDNF- and trkB-like immunoreactive neuronal perikarya could also be observed. In RVT-treated rats, BDNF-positive neuronal processes appeared more elongated and frequent than those observed in the vehicle-treated ones. Double immunostaining for BDNF and either GFAP (astrocyte marker) or Iba1 (microglia marker), carried out in selected series of brain sections from BCCAO/R+RVT rats, showed that colocalization was virtually absent, demonstrating that BDNF immunolabeling was localized to neuronal structures (Figure 4).

## 4. Discussion

In this study, we have shown that pre-treatment with a single acute dose of RVT exerts major preventive effects against the BCCAO/R-induced molecular adaptations by upregulating the relative levels of proteins involved in neuronal plasticity and synaptic adjustment in response to the tissue challenge. In particular, RVT induced in the frontal cortex (a) a general massive increase of relative protein levels of BDNF, PSA-NCAM, and Arc, the product of the immediate early gene *Arc*, in both sham and BCCAO/R conditions, and (b) an increase of trkB protein in sham-operated rats. Data obtained further extend the range of molecular markers undergoing early changes in response to a BCCAO/R protocol carried out with the same length of hypoperfusion/reperfusion used in this study [3,4,6], by supporting the evidence that the BCCAO/R-induced cerebral hypoperfusion/reperfusion is sufficient for effecting a significant decrease of BDNF and PSA-NCAM, and a significant increase of trkB protein expression, as compared to sham-operated rats.

RVT is a lipophilic molecule that is quickly metabolized following oral administration, and displays high efficacy *in vivo* [55,56,57,58]. Studies on RVT pharmacokinetics in rats indicate that it has a poor bioavailability, its plasmatic levels reaching their top 5–10 min after oral administration, with elimination half-life of 12–15 min [59]. The polyphenolic nature of RVT hampers its solubility in water (~3 mg/100 mL), as indicated by the European Pharmacopeia [60]. Nonetheless, experimental evidence showed that RVT can cross the blood-brain barrier [57,61,62,63,64] and that a robust increase of RVT concentration is detectable in the brain after intraperitoneal (i.p.) injection, reaching a peak 4 h after administration [63]. The biological characteristics of RVT have been characterized in preclinical reports and clinical trials [55] and innovative delivery strategies to improve its bioavailability, such as the complexation with liposomes [65], lipid-core nanoparticles [66], and the self-emulsifying delivery system (SEDDS) technique [67], have been designed. Thus, in studies of SEDDS characterization, it appears that the solubility profile of RVT includes emulsions in edible oils [67]. In our experimental setting, with no purpose of addressing the topic of RVT delivery efficiency, we chose sunflower oil as the vehicle, an edible oil, the molecular components of which share a similar fatty acid composition to the rat’s daily diet.

Studies carried out *in vitro* and in animal models of human pathologies demonstrated that RVT drives beneficial effects on the brain by preventing age-related neurological disorders, providing neuroprotection and stimulating synaptic plasticity [58]. Multidisciplinary studies have established a correlation between the RVT effective properties and BDNF expression [9,58], inspiring the notion that dietary intake and brain function are interrelated. Thus, RVT has been shown to trigger hippocampal neuronal plasticity by directly promoting BDNF synthesis through a complex metabolic network, including downregulation of specific micro RNAs and upregulation of the cAMP Responsive Element-Binding protein (CREB), a transcription factor strictly implicated in both physiological and pharmacological control of BDNF expression [9,37,68,69]. Further experimental data revealed that RVT increases BDNF serum concentration [70], upregulates BDNF mRNA in the rat hippocampus [35] and stimulates memory and learning through hippocampal BDNF-trkB signaling in Pb intoxicated rats [71]. Interestingly, for both RVT and BDNF, it has been suggested that they maintain stable cerebral blood flow [70], which, beyond the presence of collateral systems allowing rapid blood flow compensation, might be useful to counteract the hypoperfusion/reperfusion challenge.

In our experimental conditions, the semiquantitative analysis of frontal cortex homogenates after 30 min of BCCAO followed by 60 min of reperfusion reveals that BDNF relative protein levels are reduced compared to sham animals. To the best of our knowledge, no available studies dealt with the acute BCCAO/R-induced changes of mature BDNF protein in the frontal cortex; however, our findings are in agreement with data obtained after the onset of permanent BCCAO in both normotensive and spontaneously hypertensive rats [72], showing a decrease of BDNF protein and mRNA levels at 1 week post-surgery. Similar data, reporting virtually no changes of BDNF mRNA expression, are available in the neocortex (namely the entorhinal area) as early as 60 min after the onset of chronic BCCAO [73]. In this study, the RVT treatment induced a marked increase of the BDNF protein level in both sham and hypoperfusion/reperfusion conditions; nevertheless, did not revert the BCCAO/R-induced reduction of BDNF protein. Immunohistochemistry also showed a remarkable increase of neuronal processes in the cerebral cortex of RVT-treated rats, in keeping with data on prefrontal cortex of aged rats after chronic RVT administration [74]. As for trkB, the BCCAO/R-induced increase that we observed in vehicle-treated rats is in line with the trkB mRNA upregulation found to be induced by severe ischemia models [75,76,77,78,79,80]. We found that trkB underwent BCCAO/R-induced changes opposite to those shown by the BDNF, leading to suggest that the trkB and its ligand complement each other to confer resistance and/or reduce brain damage following the hypoperfusion/reperfusion in vehicle-treated rats. Somewhat surprisingly, this reciprocal compensation of trkB and BDNF protein expression does not follow the same scheme after RVT pre-treatment. An explanation could be that the RVT-induced stabilization of trkB protein levels may help restraining the undesirable possibility that BDNF, beside the neuroprotective role, enhances the excitatory neurotransmission [76,81]. The immunohistochemical distribution of BDNF and trkB strengthen the inferring that BDNF and trkB undergo opposite and complementary changes under the BCCAO/R. Moreover, the almost identical distribution pattern of the two markers in the frontal cortex allows considering that RVT influence is also affecting the mechanisms of BDNF-mediated trophic support. As a matter of fact, it has been shown previously that cortical BDNF immunoreactivity is localized mainly to corticofugal projecting neurons and suggested that is anterogradely transported towards the trkB containing target neurons [82,83]. However, as already proposed by tract-tracing and axotomy data [84,85], alternative local (autocrine/paracrine) actions of BDNF cannot be ruled out.

From our data it could be hypothesized that in BCCAO/R animals both RVT and BDNF may have effects that converge towards preservation of neuronal membrane, synaptic integrity, and neuroplasticity, sustaining a causality in the dynamic interplay of RVT and the neurotrophin. As already suggested in aged rats, beyond the maintenance of neuronal dendritic arborization by stimulating expression of BDNF, RVT may contrast the shrinkage of dendritic spines induced by oxidative stress [74]. In the aged brain, the increased generation of oxidative species, such as lipoperoxides, leads to cerebral oxidative stress, similarly to what observed during the BCCAO/R [4]. In these conditions, one of the most affected cellular structures is the neuronal membrane, because the lipoperoxidation processes may severely affect its plasticity at synaptic sites. We have previously shown that the BCCAO/R (performed with the same durations used in this study) increased tissue levels of endocannabinoids, palmitoylethanolamide (PEA; a ligand of PPAR-alpha), lipoperoxides, type 1 and 2 cannabinoid receptors (CB1 and CB2), and decreased brain tissue concentrations of DHA [6]. RVT pre-treatment affected the BCCAO/R-induced tissue changes by restoring DHA and augmenting PEA tissue concentrations, decreasing lipoperoxides, and increasing expression of CB receptors, and PPAR-α [4]. In parallel, RVT also increased synaptophysin in BCCAO/R rats vs. vehicle-treated ones, and markedly augmented levels of expression of post-synaptic density-95 protein (PSD-95) in BCCAO/R vs. sham rats [4].

In this study, RVT pre-treatment is also shown to induce a substantial increase of PSA-NCAM and Arc protein levels in both sham and BCCAO/R conditions. Literature data regarding RVT effects on plasticity-related molecules focus on the hippocampus and the structural remodeling occurring in learning and memory. Our data meet findings in young rat hippocampus, where PSA-NCAM expression has been shown to be upregulated by polyphenol enriched diet [86]. In this context, it is of interest that the degree of polysialylation of NCAM is linked to facilitation of BDNF-trkB signaling [44,87], and that, as already indicated above, BDNF is upregulated by RVT [35,37,38,70]. However, due to contrasting evidence showing the RVT-induced downregulation of PSA-NCAM expressing neurons in the hippocampus [88], the exact role of RVT in regulating the PSA-NCAM expression during cerebral ischemia still awaits to be clarified. As for the regulation of dendritic morphology during synapse remodeling, several coordinated signaling pathways, such as the activation of PI3 kinase/Akt/mTOR and Mitogen-associated protein kinase (MAPK) cascade, leading to CREB activation, concur to its achievement [89,90]. In parallel with the BDNF-trkB system, NCAM itself triggers an intrinsic signaling and turns on the MAPK pathway to modulate dendritic cytoskeleton [91]. Our present findings show that Arc, a key protein for the regulation of dendritic spines [42,43], is markedly increased in RVT-treated rats. Though it is unattainable to speculate whether the increased levels of Arc protein go in parallel with modulation of synaptic plasticity, it is relevant that Arc is upregulated by RVT together with the upregulation of BDNF endogenous levels. Interestingly, in basal conditions, BDNF has been shown to have a key role in enhancing the inhibitory cortical signaling, coordinating its action with endocannabinoids [92,93]. Whether the beneficial effect of RVT for the hypoperfusion/reperfusion cortical challenge may also reside in the control of Arc-induced homeostatic scaling of excitatory synapses remains to be further investigated. Conversely, the RVT-induced sparing of DHA tissue content in the BCCAO/R [4] raises also the interesting question whether, in the BCCAO/R, the restored DHA concentration is also concurring to the rise of BDNF levels, as suggested by evidence on DHA-enriched diet [94], and contributing to the plasticity of synaptic proteins [4], PSA-NCAM and Arc. Altogether, the relationship among RVT dietary intake, tissue availability of DHA, upregulation of BDNF expression, positive modulation of plasticity-related immediate early gene products, and structural neuronal proteins highlights further the dynamic interconnectivity between nutritional status and brain homeostasis [95], that appears to be of importance in the cerebral oxidative stress induced by the BCCAO/R. This functional interrelation is also evidenced by reported roles of BDNF in decreasing food consumption, regulation of glucose levels, increase of cerebral energy expenditure [96,97,98], and by implications of a deficient BDNF-trkB signaling in the onset of metabolic disorders, such as obesity [99,100].

## 5. Conclusions

To the best of our knowledge, this is the first report showing that in the frontal cortex of BCCAO/R vehicle-treated rats, BDNF and PSA-NCAM decreased, while trkB increased. RVT pre-treatment augmented all examined markers in both sham- and BCCAO/R conditions; still, a BCCAO/R-induced reduction was observed for BDNF, PSA-NCAM, and Arc, the product of the immediate early gene *Arc.* Collectively, the gathered data indicate that the RVT treatment is effective in inducing neuronal plasticity by several ways: upregulating PPARα, sparing neuronal membrane DHA, upregulating synaptic proteins synaptophysin and PSD-95 [4], increasing BDNF, PSA-NCAM and Arc in the frontal cortex of BCCAO/R rats. Additional studies are necessary to evaluate whether the RVT-induced augmentation of plasticity-related molecules is directly related to modifications of neuronal dendritic morphology, as shown in the rat prefrontal cortex [74].

The observed RVT-induced changes represent multiple aspects of plasticity that, similar to what already suggested for neurotrophins [101], guarantee the maintenance of synaptic plasticity within a functional dynamic range. In this context, the time course of these changes during longer time points of reperfusion (e.g., at 6, 12 and 24 h after BCCAO/R) would be of help in understanding how RVT may favor the synaptic plasticity events. Further studies aimed to evaluate the possible neuronal colocalization of BDNF protein, BDNF mRNA, and trkB are warranted to clarify the mechanisms of BDNF trophic support in the rat frontal cortex following the BCCAO/R. Indeed, the RVT-induced increase of endogenous BDNF levels appears a key element useful both for an adequate trophic support to hypoperfused/reperfused cerebral tissue and for its involvement in the regulation of cerebral energy balance. The results are therefore consistent with a role of RVT in preserving and/or enhancing the nervous tissue neuroplastic potential and further suggest the efficacy of dietary RVT to preserve the nervous tissue integrity and control the physiological response to the hypoperfusion/reperfusion challenge.

## Figures and Tables

**Table 1:** *F*-values and significance levels from two-way ANOVA performed on data obtained after BCCAO/R and resveratrol (RVT) pre-treatment by means of Western blot in the rat frontal cortex.

Marker	ANOVA Factors						
	BCCAO/R		RVT Treatment		RVT Treatment × BCCAO/R		
	*F*-Value	*p*-Value	*F*-Value	*p*-Value	*F*-Value	*p*-Value	DF
**BDNF**	26.34	<0.0001	44.49	<0.0001	1.707	ns	1, 18
**trkB**	2.389	ns	9.461	0.0065	8.672	0.0087	1, 18
**PSA-NCAM**	19.64	0.0003	22.09	0.0002	0.0003	ns	1, 18
**Arc**	1.179	ns	63.86	<0.0001	2.012	ns	1, 18

Arc, activity-regulated cytoskeleton-associated protein; BDNF, brain-derived neurotrophic factor; PSA-NCAM, polysialylated neural cell-adhesion molecule; trkB, tyrosine kinase receptor B. DF, degrees of freedom; ns: not significant.

## Abbreviations

Arc	Activity-regulated cytoskeleton-associated protein
BCCAO/R	bilateral common carotid artery occlusion followed by reperfusion
BDNF	Brain-derived neurotrophic factor
CB	cannabinoid receptor
COX-2	cyclooxygenase-2
CREB	cAMP Responsive Element-Binding protein
DHA	docosahexaenoic acid
eCBs	endocannabinoids
GAPDH	Glyceraldehyde-3-Phosphate Dehydrogenase
GFAP	Glial fibrillary acidic protein
PBS	Phosphate-buffered saline
PEA	palmitoylethanolamide
PPAR-α	peroxisome-proliferator activated receptor-α
PSA-NCAM	Polysialylated neural cell-adhesion molecule
PSD-95	post-synaptic density protein-95
RVT	resveratrol
SDS	PAGE Sodium dodecyl sulphate
TBS	Tris base, Sodium chloride, Tween 2
trkB	Tyrosine kinase receptor B
